# Determination of Odor Air Quality Index (*OAQI*_I_) Using Gas Sensor Matrix

**DOI:** 10.3390/molecules27134180

**Published:** 2022-06-29

**Authors:** Dominik Dobrzyniewski, Bartosz Szulczyński, Jacek Gębicki

**Affiliations:** Department of Process Engineering and Chemical Technology, Faculty of Chemistry, Gdansk University of Technology, 11/12 G, Narutowicza Str., 80-233 Gdansk, Poland; domdobrz@student.pg.edu.pl (D.D.); jacek.gebicki@pg.edu.pl (J.G.)

**Keywords:** gas sensors, sensor matrix, odor concentration, field olfactometry, odor index

## Abstract

This article presents a new way to determine odor nuisance based on the proposed odor air quality index ($OAQI_{I}$), using an instrumental method. This indicator relates the most important odor features, such as intensity, hedonic tone and odor concentration. The research was conducted at the compost screening yard of the municipal treatment plant in Central Poland, on which a self-constructed gas sensor array was placed. It consisted of five commercially available gas sensors: three metal oxide semiconductor (MOS) chemical sensors and two electrochemical ones. To calibrate and validate the matrix, odor concentrations were determined within the composting yard using the field olfactometry technique. Five mathematical models (e.g., multiple linear regression and principal component regression) were used as calibration methods. Two methods were used to extract signals from the matrix: maximum signal values from individual sensors and the logarithm of the ratio of the maximum signal to the sensor baseline. The developed models were used to determine the predicted odor concentrations. The selection of the optimal model was based on the compatibility with olfactometric measurements, taking the mean square error as a criterion and their accordance with the proposed $OAQI_{I}$. For the first method of extracting signals from the matrix, the best model was characterized by RMSE equal to 8.092 and consistency in indices at the level of 0.85. In the case of the logarithmic approach, these values were 4.220 and 0.98, respectively. The obtained results allow to conclude that gas sensor arrays can be successfully used for air quality monitoring; however, the key issues are data processing and the selection of an appropriate mathematical model.

## 1. Introduction

The dynamic development of modern civilization and the increasing level of consumption generate the problem of a huge amount of waste, which is a deadly threat to the environment and human health. Currently, in addition to depositing waste in municipal landfills, great emphasis is placed on the issues related to their sorting, recycling and composting. However, all these processes can cause some negative consequences. One of them is the introduction of solid, liquid or gaseous pollutants to the natural environment, which poison the soil, water and the atmosphere [1,2,3,4,5,6,7]. Among gaseous air pollutants, there is a group of compounds characterized additionally by odor nuisance. This group includes the volatile components of air pollutants that have toxic properties, are detectable at relatively low concentrations, and cause undesirable odor sensations. Volatile chemicals that make up the gases emitted into the atmosphere can be divided into volatile organic compounds (VOCs) and volatile inorganic compounds (VICs).

Odorants emitted to the atmosphere may be of natural or anthropogenic origin. Sources of odor-forming substances, which are a side effect of human activity, are mainly the chemical industry [8], food industry [9], fuel industry [10], municipal sewage treatment plants [11,12], fragrance industry [13,14], composting facilities [15,16,17], municipal landfills [18,19] and swine farming and breeding [20,21,22]. The nature of odors generated by municipal waste management facilities depends on many factors, such as type of waste, method of storage, compost production techniques, etc. However, all these facilities emit pollutants of similar qualitative composition, and the odor nuisance is mainly related to the presence of the following compounds: disulfides, amines, sulfides, ammonia, thiols, alcohols, carboxylic acids, aldehydes, ketones, phenols, and aliphatic amines [13,19,23,24,25,26,27,28,29,30,31,32,33,34,35]. The ranges of olfactory detection threshold and the perceived odor character for exemplary odorants are presented in Table 1.

In order to properly assess the environmental effects of odorants emitted into the atmosphere, measurement techniques are used that allow for both quantitative and qualitative analyses of the composition of polluted air. There are two basic techniques for determining odor nuisance: sensory techniques and instrumental (analytical) ones. In sensory techniques, such as dynamic olfactometry or field olfactometry, the role of the detector is played by the human sense of smell. Analytical techniques focus primarily on the use of gas chromatography or gas sensor arrays that are gaining in popularity. They enable a holistic analysis of the composition of the gas mixture without dividing it into individual components, and the analysis time is much shorter. Gas sensor matrices also provide the ability to monitor industrial processes continuously without any sampling. Obviously, in terms of the accuracy of the obtained results, they do not match the chromatographic techniques; however, in cases requiring, for example, quick reaction of industrial facilities, staff sensor arrays are an excellent alternative. Their high application potential has been proven for processes such as monitoring of biofiltration [40,41,42,43,44,45,46], monitoring of methane reforming process [47], monitoring of sanitary conditions [48], monitoring and identification of air pollutants and hazardous substances [49,50,51,52], quality assessment of food products [53,54,55,56,57,58], detection of drugs and explosives [59,60,61], in medicine as a non-invasive diagnostics systems for analysis of breath or urine [62,63,64,65,66], to control indoor air quality [67,68,69] or in the perfume industry to confirm the authenticity of products [55,70].

The main difference between the sensory and instrumental techniques is that the analytical measurements focus on individual odorants, while sensory measurements are mainly concerned with general odor sensations. However, in order to develop innovative and effective odor quality monitoring systems, a complementary and integrated approach based on the synergy of their operation is necessary. Attempts to combine the advantages of both groups of techniques were already being made, for example in the form of gas chromatography coupled with olfactometry (GC-O) [71,72,73,74,75] or by trying to demonstrate the validity of using sensor arrays for odor monitoring by comparing them with gas chromatography–mass spectrometry (GC-MS) [30,76,77].

As mentioned before, determining the impact of the industrial or municipal plants on the odor nuisance of a given area requires taking into account several key parameters of the smell. Among them, the following should be particularly distinguished:

- Hedonic tone—pleasant or unpleasant sensations during inhalation of a gaseous mixture;
- Odor intensity—relative strength of the odor stimulus induced by a particular odorant;
- Odor threshold—the minimum concentration of a substance at which most test subjects can identify the odor.

These three parameters are crucial to establish the relationship between odor concentration and the odor nuisance, and they make it possible to better understand the interaction between emitted odorants. For this reason, in order to estimate the odor nuisance of a municipal plant, it is necessary to use indices and indexes embodying all of them. So far, several indexes have already been proposed and presented, showing the correlation between sensorial and analytical characterization of odors. The most common indices used to assess odor nuisance include the following:

- Specific odor emission rate (SOER)
  (1) $$SOER=\frac{Q_{air}*C_{od}}{A_{base}}$$
  where:
  $A_{base}$—base area [$m^{2}$]
  $C_{od}$—odor concentration [$\frac{ou}{m_{3}}$]
  $Q_{air}$—air flow [$\frac{m^{3}}{s}$]
- Odor emission rate (OER)
  (2) $$OER=SOER*A_{landfill}$$
  where:
  $A_{landfill}$—emitting surface of the considered area [$m^{2}$]
- Odor emission factor (OEF)
  (3) $$OEF=\frac{OER}{AI}$$
  where:
  *AI*—representative “activity index” of the plant. It can be represented by e.g., plant capacity, landfill surface, mass of processed material or yearly treatment capacity [78,79,80,81,82].
- Analytical odor index (AOI)/odor activity value (OAV)
  (4) $$AOI/OAV=\sum_{i=1}^{n}\left(\frac{C_{y}}{OT_{y}}\right)$$
  where:
  $C_{y}$—concentration of y-th substances detected by GC techniques [ppm]
  $OT_{y}$—relative Odor Threshold [ppm]
- Sensorial odor index (SOI)
  (5) $$SOI_{i}=\frac{U_{i}}{U_{s}}$$
  where:
  $U_{i}$—concentration of odor at i-th sampling point [$\frac{ou}{m_{3}}$]
  $U_{s}$—standard concentration of odor detectable by sensorial analysis [$\frac{ou}{m_{3}}$]
- Odor index (OI)
  (6) $$OI=10log(C_{od})$$
  where:
  $C_{od}$—odor concentration [$\frac{ou}{m_{3}}$]
- Odor annoyance index (OAI)
  (7) $$OAI=\frac{1}{N_{t}}\sum_{i=1}^{n}w_{i}N_{i}$$
  where:
  $N_{t}$—total number of observations for the investigated zone or period
  $N_{i}$—number of observations corresponding to the odor rating *i* (*i* = 0–6)
  $w_{i}$—corresponding weighting factors

Table 2 provides examples of industrial and municipal facilities for which an odor nuisance assessment was conducted using the indicators listed above.

This article presents a proposal for a new odor air quality index ($OAQI_{I}$) which was determined on the basis of the emission measurements of odorants in the environment. The basic assumption of the proposed index is the ability to simultaneously interpret all of the major odor features: hedonic tone, intensity and odor concentrations. In other words, based on the proposed index, the determination of one of these parameters enables the estimation of the others. The first two parameters were determined using parametric sensory measurements (numerical scales). The odor load that was released into the air as a result of the emission (odor concentration) was measured using field olfactometry and a gas sensor array.

The application of a gas sensor matrix to estimate odor concentration would enable air quality monitoring in terms of odor nuisance continuously (online mode) with easy and quick access to the collected data. Such usages of sensor arrays have already been reported in the literature, for example, for quantitative evaluation of pond odors from piggeries [91]. Odor concentrations were determined by dynamic olfactometry, and an artificial neural network (ANN) was used to correlate them with the signals received from sensors. It has been shown that a sensor matrix connected to a trained ANN is able to predict odor concentrations with a low root mean square error (RMSE) and a coefficient of determination ($R^{2}$) between olfactometric measurements and the predicted concentrations in unknown samples at the level of 0.92.

Another example could be sensor-based measurements of odor concentrations in grasslands after using cattle slurry on them [92]. In this study, a comparison was made between a commercially available gas sensor array and self-built device. In order to determine the odor concentrations in the analyzed samples, dynamic olfactometry (DO) was used again. On the basis of the principal component analysis (PCA) of actual sensor response patterns, linear relationships were established between odor concentrations and average response of conductive polymer type sensors. A single line fitted to the data from all experiments had a percentage of the variance accounted for 59% and 62% depending on the device.

In other literature reports, the possibility of using sensor arrays for continuous monitoring of odors from wastewater treatment plant or composting plant (WWTP) was investigated [93,94]. In these works, a lot of attention was paid to the selection of appropriate identification methods, definitions and optimization of the process of creating a dataset for training the sensor array to enhance its ability to correctly estimate odor concentrations. In addition, qualitative classification of the analyzed samples was performed using three different sensor arrays, consisting of six metal oxide semiconductor (MOS) type sensors each. The application of this type of sensors had made the devices sensitive to many groups of volatile chemicals, which is decent in terms of the complex gas mixtures found in this type of plants. After selecting the optimal algorithm, a qualitative classification accuracy of 96.4% and a correlation between actual and predicted odor concentrations expressed by a determination coefficient of 0.90172 were obtained.

The study conducted in [95] also assessed the odors from WWTP with the use of 12 conductive polypyrrole polymer sensors. As a statistical technique for data analysis, canonical discriminator (CD) and canonical correlation (CC) were used, which are similar to the assumptions of PCA, except that CC is the correlation that is maximized instead of variance. Linear relationships were created between odor concentrations (determined by DO) and explanatory variables (sensor signals). The research was carried out in several WWTPs in an attempt to create and demonstrate one generalized relationship between sensor array response and odor concentration from such facilities. It was noted that such a correlation is extremely difficult to achieve; however, when the approach was changed and the odor concentrations from individual treatment plants were analyzed, much stronger relationships were obtained.

Sensor matrices applications for continuous odor monitoring in poultry are also known [96]. The approach in this work is similar to the previously cited studies and involves determining odor concentrations using DO and modeling the correlation between them and a sensor array composed of 24 MOS type detectors. Data were collected continuously and due to the large number of them, PCA was applied to reduce the multidimensionality of the dataset. Then, a linear model for odor concentrations prediction was developed using partial least square regression (PLSR). Sensor resistances were used as input to the model. RMSE and root-mean square error of cross validation (RMSECV) were used to evaluate the accuracy of the prepared model. Their values were 179.9 and 182.23, respectively, which, with the coefficient of determination of the calibration curve at the level of 0.94, confirmed that it is possible to create a mathematical model that, when implemented in a sensor array, will enable the real-time measurements of odor concentrations.

All these studies demonstrated the possibility of using gas sensor arrays to predict the odor concentrations in various plants associated as sources of odor emissions. Additionally, all of them used dynamic olfactometry to determine the odor concentrations of the analyzed samples. The undoubted advantage of this method is that it is only one that is fully standardized, but on the other hand, a sampling stage is required, which is always troublesome, as well as the need for a trained team of assessors. Another thing they have in common is the development of linear predictive mathematical models of odor concentrations based on various methods of analyzing sensor array data. The purpose of this research was to develop such a predictive model for a self-constructed gas sensor matrix, but as a reference technique, field olfactometry was used. The constructed gas sensor array was located in the compost screening yard and consisted of commercially available chemical sensors. The article describes the calibration of the prepared matrix using five mathematical models and the subsequent validation of the models developed. The calibration and validation of the models were carried out on the basis of periodic olfactometric measurements; therefore, the odor concentration was a dependent variable (model response), while the signals from the sensors were treated as explanatory variables.

The applicability and accuracy of the prepared models with respect to olfactometric measurements and their compatibility with the proposed $OAQI_{I}$ were evaluated. The main presumption of the suggested index was to correlate and combine the predicted odor concentrations with other air quality parameters, which may be more transparent to the majority of the society unfamiliar with the concept of odor concentrations.

## 2. Materials and Methods

**Figure 1:**
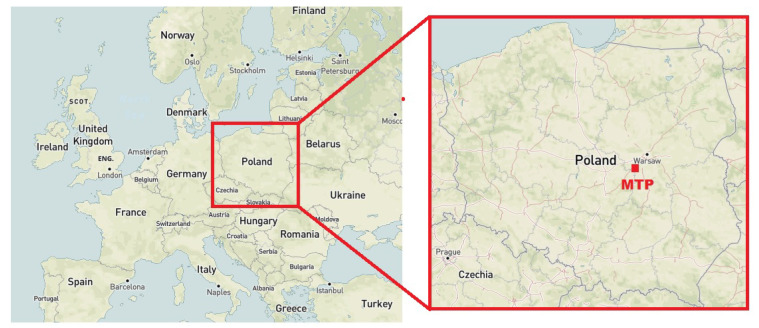
A map showing the location of the municipal treatment plant where the research was conducted.

The measurements and research were conducted at a municipal solid waste treatment plant in Central Poland (Figure 1).

**Figure 2:**
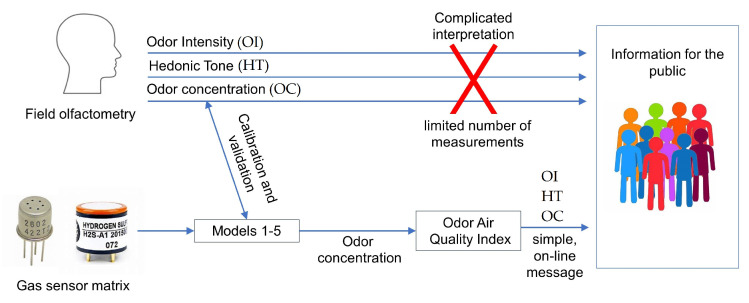
The concept of the conducted research.

The sensor array was placed in the compost screening yard. This localization provided episodic high odor concentrations associated with the operation of the compost screening process, while during the rest of the time, the odor concentrations were relatively low. Periodically, the field olfactometry measurements were carried out at the sensor matrix location using a Nasal Ranger olfactometer. Data processing, analysis and other calculations were performed using RStudio Desktop (v.1.0.143) software. The concept of the conducted research is presented graphically in the Figure 2.

### 2.1. Field Olfactometry

The gas sensor matrix was calibrated using the Nasal Ranger field olfactometers (St. Croix Sensory, Inc., Stillwater, MN, USA). This approach was aimed at linking signals from gas sensors with specific odor concentrations. The olfactometric measurements were carried out each time with the participation of at least two people. During the analysis, all recommendations regarding the measurement procedure were followed. The result of the field olfactometry measurement is the numerical value of the dilution to olfactory threshold (D/T) ratio, read from the dilution dial on the front of the olfactometer. This parameter should be interpreted as the number of dilutions needed to make the odor of contaminated air undetectable [97]. The D/T values correspond, after conversion, to the $Z_{ITE}$ value (individual estimation of the dilution to threshold) defined in EN 13725. $Z_{ITE}$ was calculated for each team member separately according to the relationships:

(8) $$Z_{YES}={\left(D/T\right)}_{YES}+1$$

(9) $$Z_{NO}={\left(D/T\right)}_{NO}+1$$

(10) $$Z_{ITE}=\sqrt{Z_{YES}*Z_{NO}}$$

where:

$Z_{YES}$—the dilution level at which the odor is detectable

$Z_{NO}$—the dilution level at which the odor is undetectable

The odor concentration value was calculated as the geometric mean of the set of individual estimates collected by the team.

(11) $$C_{od}=\sqrt[{n}]{\prod_{i=1}^{n}Z_{{ITE}_{n}}}$$

where:

*n*—number of people participating in olfactometric measurements

In total, 60 series of olfactometric measurements were performed, which were divided into three stages. In each stage, 20 series were performed, divided equally into two measurement days. The first 20 measurements were taken in September 2021 (autumn period), the second phase was conducted in December 2021 (winter period) and the third in March 2022 (spring period). The first two stages were used to calibrate the matrix, and the third one was used to validate the proposed models. The field olfactometry measurements taken in April 2022 were used to check the reliability of odor concentrations returned by the models, with the actual condition occurring at the site.

### 2.2. Sensory Matrix Development and Measurement

Five commercially available chemical gas sensors were chosen for operation in the constructed sensor array. Three metal oxide semiconductor sensors (TGS2602, TGS2603 and TGS2612) and two electrochemical sensors ($NH_{3}$ and $H_{2}S$) were selected. The basic characteristics of the selected sensors are presented in Table 3.

Since the constructed gas sensor array was placed on the compost screening site, the gas sensors were selected to best match the characteristics of the investigated environment. Therefore, two electrochemical sensors were used that were able to detect hydrogen sulfide and ammonia, gases whose concentrations were expected to exceed their olfactory threshold. The other sensors were non-selective semiconductor sensors that respond to organic air pollutants. Lack of selectivity does not render these sensors useless because grouping them into an array makes the resulting signal multidimensional. Additionally, these studies did not focus on precise determination of concentrations of individual odorants, but only on the evaluation of odor nuisance, which is caused by the synergic action of all compounds forming a gaseous mixture.

However, the high sensitivity of TGS-type sensors to the presence of methane must be taken into account. This gas belongs to a group of compounds called aliphatic hydrocarbons and is known to be colorless and odorless. For this reason, its presence in the air will not affect the level of odor nuisance. This means that methane will not be detected during field olfactometry measurements, but may significantly affect the signals received from the gas sensor array. Therefore, during the analysis of the collected signals from the matrix, those that were represented by low odor concentrations and incomparably high values from analog-to-digital converters were rejected.

### 2.3. Signals’ Features Extraction

All analog electric signals obtained from the sensors were digitized using ADS1115 16-bit analog-to-digital converter (Texas Instruments, Dallas, TX, USA) and saved on a personal computer. In this paper, two approaches are proposed for signal extraction from a sensor array:

1. The maximum signal value—$S_{max}$;
2. Logarithm of the ratio of the maximum signal value to the baseline—$log_{10}\left(\frac{S_{max}}{S_{0}}\right)$.

**Figure 3:**
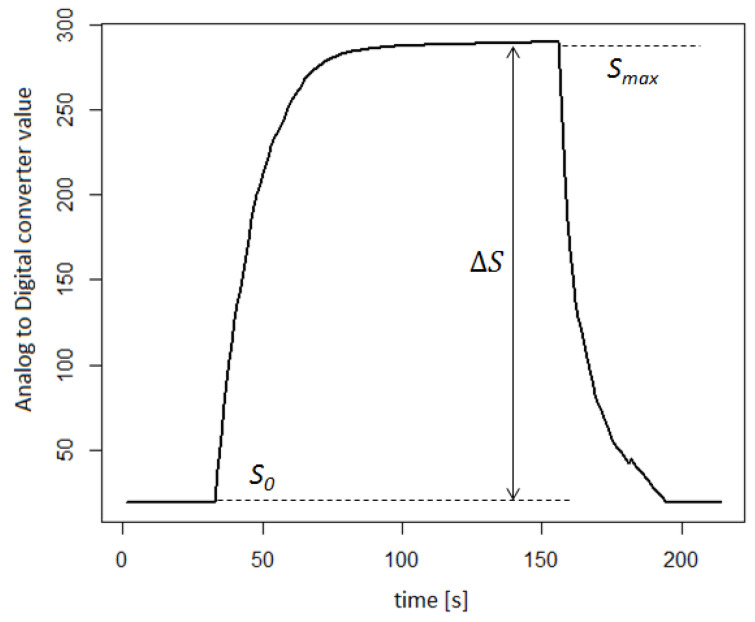
Example of the TGS2602 sensor response with marked signal parameters used in further data analysis.

An example of the sensor response is shown in Figure 3. The baseline of each sensor used was determined using synthetic air. The logarithmic approach is to reflect the psychophysical models of odor perception (Weber–Fechner law).

The second of the described methods of data extraction from the matrix reflects the logarithmic unit of measure of quotient quantities, defined as Bel (*B*). Biological systems, including the human sense of smell, react to external stimuli according to Weber–Fechner’s law, that is, in a non-linear way. For this reason, it is reasonable to use the quotient–logarithmic measure to describe all kinds of psychophysical phenomena.

In the calculations performed, it was decided to use the derivative of the Bel unit, which is the decibel (*dB*). This unit is one-tenth of a Bel (*B*) and is used to compare quantities that vary linearly over a wide range. The signal from each sensor used in the array was therefore converted to *dB* according to the following relation:

(12) $$S_{dB}=10log_{10}\left(\frac{S_{max}}{S_{0}}\right)$$

where:

$S_{0}$—sensor baseline

$S_{dB}$—maximum sensor signal value in *dB*

$S_{max}$—maximum sensor signal value

### 2.4. Mathematical Models for Odor Concentration Determination

A total of 60 olfactometric measurements was made, which allowed to distinguish a training set (40 measurements) and a test set (20 measurements). Based on the training data, the mathematical calibration models were drawn up linking the odor concentrations determined olfactometrically with sensor signals operating in the matrix. It was decided to use the following statistical techniques that use explanatory variables (senor signals) to predict the outcome of the response variable (odor concentration):

- Model 1: Multiple Linear Regression (MLR)
  *MLR* is a statistical technique based on the generation of a linear relationship between the independent variables (sensor signals) and the dependent variable (odor concentration). It is important that the explanatory variables are not correlated with each other and their number is smaller than the number of observations (measurements) made. The general equation is as follows:
  (13) $$C_{od}=a_{0}+\sum_{i=1}^{n}\left(a_{i}*S_{i}\right)$$
  where:
  $a_{0}$—intercept
  $a_{i}$—regression coefficients
  $S_{i}$—sensor signal (depending on the method of signal’s features extraction)
  *n*—number of explanatory variables
- Model 2: Principal Component Regression (PCR)
  *PCR* allows for the selection of explanatory variables that have a significant impact on the value of the dependent variable; these are called principal components (PC). PC selection is performed using principal component analysis (PCA) and then the MLR model is created in which $PC_{s}$ are used as explanatory variables. Formally, PCR is defined as follows:
  (14) $$C_{od}=a_{0}+\sum_{i=1}^{n}\left(a_{i}*PC_{i}\right)$$
  where:
  $a_{0}$—intercept
  $a_{i}$—regression coefficients
  $PC_{i}$—principal components
  *n*—number of principal components
- Model 3: Stevens’ power law with a single sensor signal as a power exponent
  Signals from individual sensors were used as an exponent of the proposed relationship. The model best suited to the training dataset was selected based on the value of the coefficient of determination ($R^{2}$). Despite the fact that this coefficient ignores the adjustment of the model to data outside the training set, it is one of the basic measures of the quality of the model matching to this data set. The odor concentration was determined according to the following formula:
  (15) $$C_{od}=ab^{S}$$
  where:
  *a*—first model coefficient
  *b*—second model coefficient
  *S*—sensor signal (depending on the method of signal’s features extraction)
- Model 4: Stevens’ power law with the geometric mean of the sensor signals as a power exponent
  This approach was used only for the second method of extracting signals from the sensor array $\left(S_{dB}\right)$. The geometric mean of the signals from the individual sensors was used as the exponent of the function in accordance with relation
  (16) $$C_{od}=ab^{\sqrt[{n}]{\prod_{i=1}^{n}{S_{dB}}_{i}}}$$
  where:
  *a*—first model coefficient
  *b*—second model coefficient
  $S_{{dB}_{i}}$—sensor signal value in dB
  *n*—number of sensor in the matrix
- Model 5: Stevens’ power law combined with Principal Component Analysis (PCA)
  The first principal component $\left(PC_{1}\right)$ of the dataset was used as the exponent of the proposed function. $PC_{1}$ was calculated using PCA and performing mean centering and scaling to unit variance. It shows the direction of the maximum variance in the data and best approximates the data in least squares terms. The odor concentration was computed as follows:
  (17) $$C_{od}=ab^{PC_{1}}$$
  where:
  *a*—first model coefficient
  *b*—second model coefficient
  $PC_{1}$—first principal component

The standard approach used to analyze data obtained from chemical sensor arrays was used to develop Model 1 and Model 2. Both MLR and PCR are among the most commonly used statistical methods to predict the value of the dependent variable (odor concentration) using the values of the explanatory variables (sensor signals). Model 3, Model 4, and Model 5 are hybrid models that combine Stevens’ psychophysical law with different ways of analyzing the signals received from the sensor array. These models are intended to resemble perceptual models of stimulus experience (sensation), where different, constant values assigned to a particular continuum are used as a power exponent. For the proposed models, sensor signals were introduced in place of this empirical constant after several different methods of analyzing the data obtained from the matrix.

Once the calibration models were prepared, they were validated on the basis of the data test set. To assess the compliance, usefulness and accuracy of the prepared (trained) models with the olfactometric measurements, the root mean square error (RMSE) was used. This is one of the most common methods to determine model error in predicting output variables. RMSE was calculated according to the following formula:

(18) $$\mathrm{RMSE}=\sqrt{\sum_{i=1}^{n}\frac{{\left(x_{i}-y_{i}\right)}^{2}}{n}}$$

where:

$x_{i}$—odor concentration determined from olfactometric measurements

$y_{i}$—odor concentration determined from gas sensor array

*n*—number of olfactometric measurements

Mathematically, RMSE is the Euclidean distance between a vector of observed values and a vector of predicted values multiplied by the square root of the inverse of the number of observations. In other words, the lower the RMSE values, the better the model is at predicting the observed data. Higher RMSE values should be interpreted as the model failing to account for important features underlying the data.

### 2.5. Odor Air Quality Index

**Figure 4:**
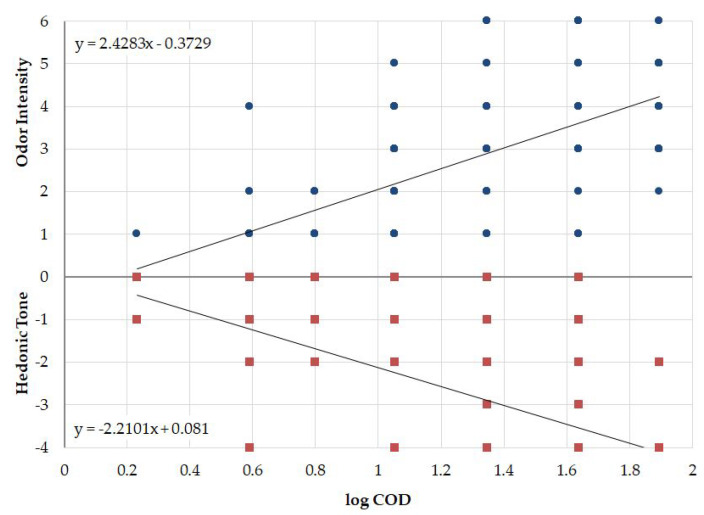
Dependence of odor intensity and hedonic tone on odor concentration carried out at the municipal treatment plant.

The proposed odor air quality index ($OAQI_{I}$) scale is based on the odor features: odor intensity and hedonic tone. On the basis of 240 measurements carried out at the municipal treatment plant, during which the odor intensity, its hedonic tone and odor concentration were assessed, a plot (Figure 4) was prepared of the dependence of the odor intensity and hedonic tone on the logarithm of the odor concentration (according to Weber–Fechner’s law, this relationship should be linear). The obtained equations were used to determine the odor concentrations corresponding to the values of odor intensity and hedonic tone.

Appropriate odor concentration ranges were assigned to the determined indexes in accordance with Table 4.

The validation of the models in terms of compliance in the $OAQI_{I}$ was assessed using the accordance parameter, according to the following formula:

(19) $$Accordance=\frac{n_{correct}}{N}$$

where:

*N*—total number of measurements

$n_{correct}$—the number of measurements classified by both the model and the olfactometric measurements to the same index scale.

## 3. Results

### 3.1. Models Development

**Figure 5:**
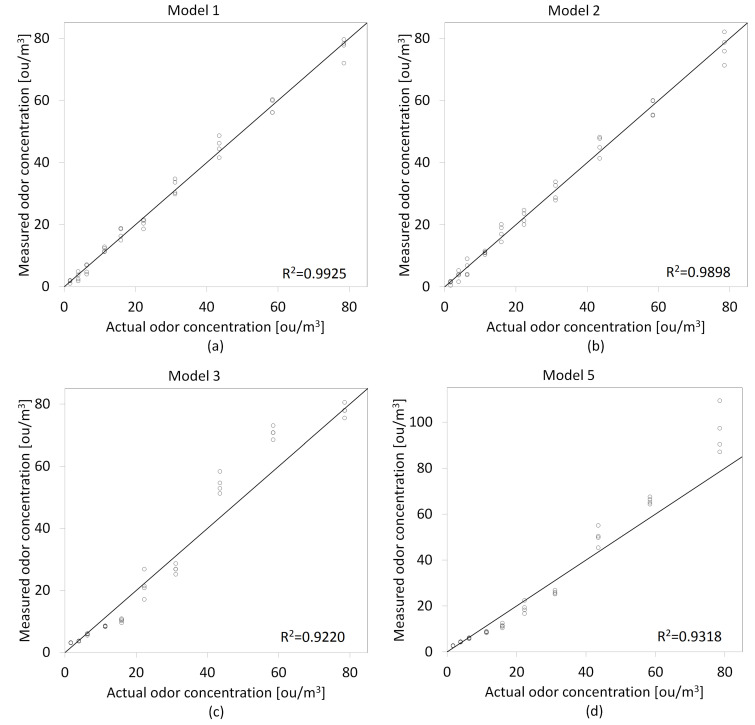
Comparison scatter plots of odor concentrations prediction models prepared using the maximum signal value—$S_{max}$ as an independent variable: (**a**) Model 1, (**b**) Model 2, (**c**) Model 3, (**d**) Model 5.

**Figure 6:**
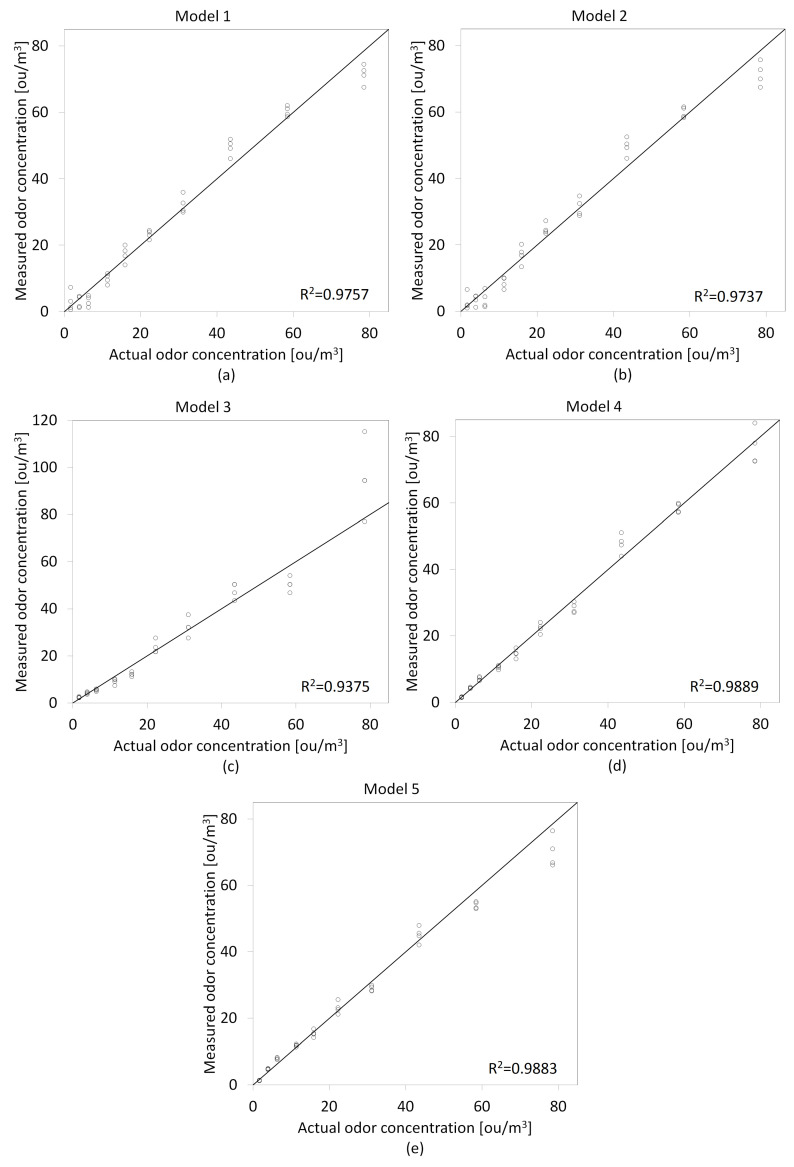
Comparison scatter plots of odor concentrations prediction models prepared using signal value in *dB*-$S_{dB}$ as an independent variable: (**a**) Model 1, (**b**) Model 2, (**c**) Model 3, (**d**) Model 4, (**e**) Model 5.

In the first stage of the conducted research, the calibration models were prepared based on the recorded responses from the gas sensor array and olfactometric measurements (training dataset). Four calibration models were prepared using the maximum signals values from individual sensors and five using a logarithmic approach to extract signals from the matrix. Comparison scatter plots of the prepared models are shown in Figure 5 and Figure 6. For Model 3, the TGS2602 sensor signal was used as a function exponent for the first method of extracting signals from the sensor array ($S_{max}$), and the ammonia sensor signal for the second method of acquiring signals from the matrix ($S_{dB}$). It was dictated by obtaining the highest values of the coefficient of determination $(R^{2})$ with the use of signals from these sensors.

### 3.2. Models Validation

**Figure 7:**
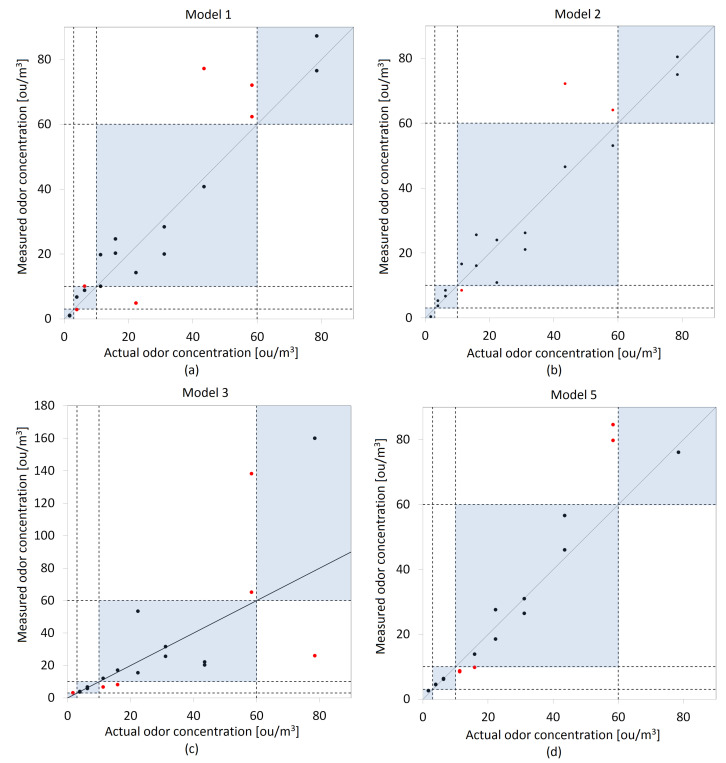
Validation plots of proposed models—actual and determined odor concentrations based on the maximum signal value—$S_{max}$ as an independent variable: (**a**) Model 1, (**b**) Model 2, (**c**) Model 3, (**d**) Model 5.

**Figure 8:**
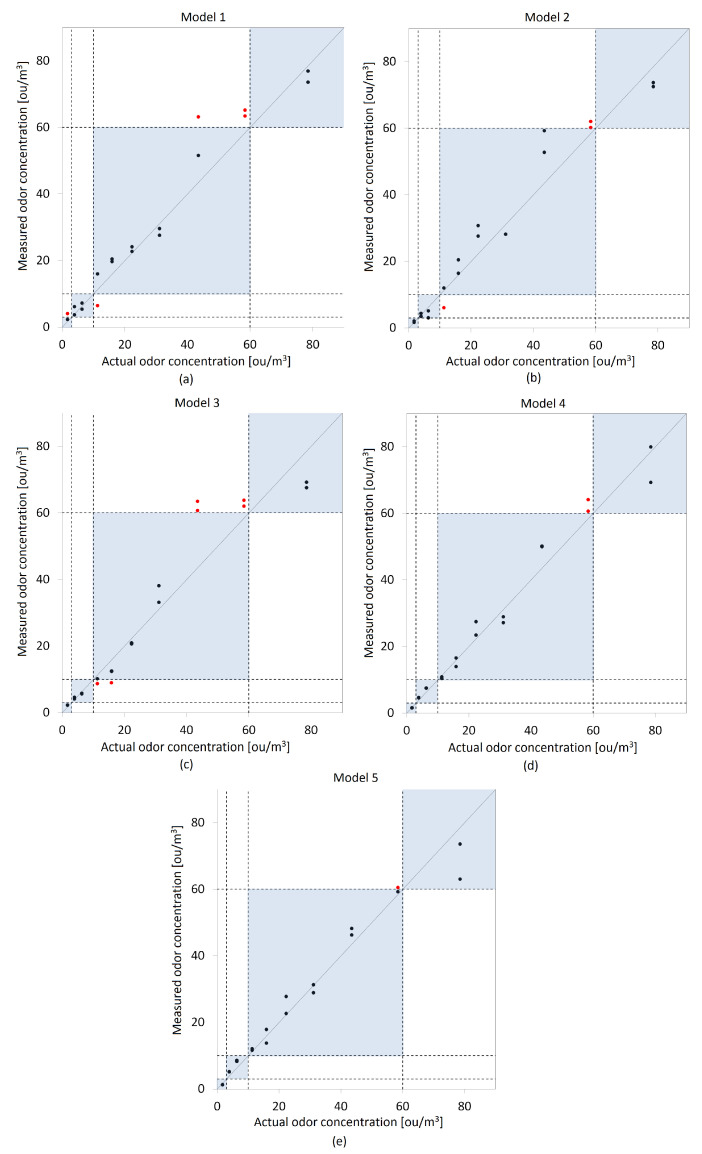
Validation plots of proposed models—actual and determined odor concentrations based on signal value in *dB*-$S_{dB}$ as an independent variable: (**a**) Model 1, (**b**) Model 2, (**c**) Model 3, (**d**) Model 4, (**e**) Model 5.

In the next stage of the research, the prepared models were validated. The validation of these models were performed on the basis of correlation plots. Figure 7 and Figure 8 present the odor concentrations determination using a gas sensor matrix and test set of olfactometric measurements for both methods of extracting signals from the matrix.

Validation charts presented in Figure 7 and Figure 8 were prepared on the basis of an olfactometric data test set (stage three of the measurements campaign). The blue rectangles on the charts reflect the proposed odor air quality index scale presented in Table 4. The black and red points in the graphs correspond to the predicted odor concentrations. The points marked in black are within the appropriate scale ranges of the proposed index, while those marked in red exceed the adopted ranges.

Root mean square error (RMSE) was chosen as the numerical tool for the selection of the optimal model in terms of matching the predicted odor concentrations to the actual results of olfactometric measurements. The adaptation of individual models was also determined on the basis of the proposed $OAQI_{I}$. A parameter called *"accordance"* was established, which reported the percentage of the odor concentrations determined by the mathematical models that coincided with the actual values within the proposed concordance intervals. Table 5 presents the results of these two parameters calculated for the prepared models.

Based on the table above, it was found that when using the maximum sensor signals as an explanatory variable in the models, the best fit to the actual odor concentration values is Model 2, i.e., principal component regression (PCR). It is characterized by both the lowest RMSE and the highest accordance value. If the $S_{dB}$ is used as the independent variable, the selection of the optimal model is somewhat more difficult. All of them are characterized by similar RMSE values, but for Model 4 and Model 5, they are the lowest. As can be seen, Model 4 has a slightly lower RMSE than Model 5, but the difference in accordance makes the latter appear more optimal.

**Figure 9:**
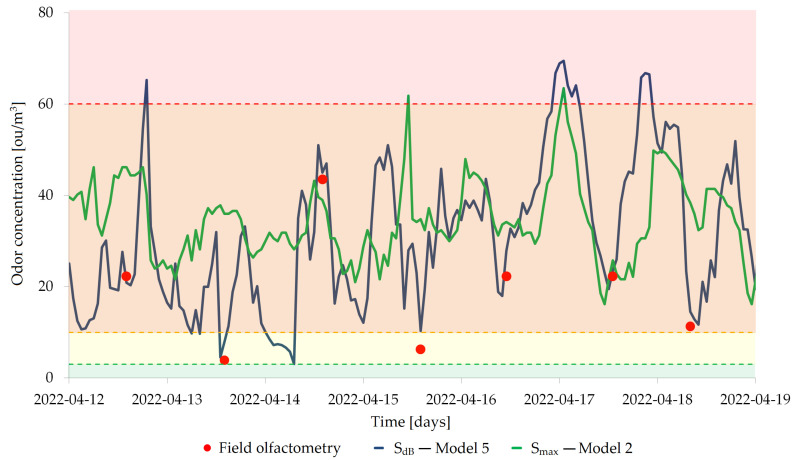
Weekly changes in odor concentrations at the compost screening site calculated using the best model developed using the maximum signal value and signal value in *dB* compared with field olfactometry measurements.

In the final stage of the study, after selecting the optimal model for both sensor array signals extraction methods, the changes in odor concentrations at the compost screening site were recorded. Figure 9 shows weekly courses of changes in the odor concentration within the compost yard for both models. Moreover, during these seven days, olfactometric measurements were performed daily at the municipal solid waste treatment plant. This was to be used to verify and possibly confirm or deny the usefulness of the developed models and the constructed sensor array for continuous monitoring of odorous air quality on the premises of the plant. On the basis of the presented graph, it can be seen that Model 5 based on $S_{dB}$ presents a better fit to the results of the field olfactometry (RMSE=3.0) than Model 2 based on $S_{max}$ (RMSE=26.5).

## 4. Discussion

This article presents a new way to determine odor nuisance based on the proposed odor air quality index ($OAQI_{I}$). During its development, odor features, such as hedonic tone, odor intensity and odor concentration, were taken into account. A custom-built sensor array consisting of five commercially available chemical sensors (three metal oxide semiconductor sensors and two electrochemical) was used to determine odor concentrations in the compost screening yard at the municipal solid waste treatment plant. In order to calibrate the sensor array, odor concentrations were measured on the MSWTP site near its location using field olfactometry. This allowed a correlation to be found between the multidimensional output signal from the sensor array and the actual odor concentration. Five statistical mathematical models were used for two methods of extracting signals from the sensor matrix. In the first approach, the maximum value of the signal from individual sensors $\left(S_{max}\right)$ was used as independent variables in the developed models. In the second one, the logarithmic approach was used $\left(S_{dB}\right)$, determining the ratio of the maximum signal from the sensor to its baseline. The selection of the optimal model was made on the basis of two parameters: root mean square error (RMSE) as the benchmark determining the compatibility of the prepared models with the olfactometric measurements, and the criterion called accordance which allowed for the validation of the models in terms of compliance in the $OAQI_{I}$.

The conducted research showed that when using the maximum value of the signal from the sensors, Model 2 was characterized by the best fit in relation to the real measurements. It achieved the lowest RMSE value in its group ($S_{max}$), amounting to 8.092, and the highest compliance in the proposed $OAQI_{I}$, equal to 85%. Using the second method of extracting data from the sensor matrix, the best model turned out to be Model 5, which is a hybrid model combining Stevens’ power law with one of the basic methods of multivariate data analysis, which was principal component analysis (PCA). The RMSE thus obtained was 4.220, and the index compliance level reached as much as 98%. It should be noted that among the model in this group ($S_{dB}$), the lowest RMSE value was obtained for Model 4 (3.667), which used the geometric mean of the signals from the sensors installed in the array as a exponent in Stevens’ power law. However, compared to the Model 5, it was characterized by a lower compliance in the proposed $OAQI_{I}$, which was 90%. The difference in the prediction of odor concentration and its actual value between these two models was not significant enough to compensate for the difference of eight percentage points in compliance with the $OAQI_{I}$ index. Therefore, Model 5 was selected as the optimal model from the $S_{dB}$ group.

In both the $S_{max}$ and $S_{dB}$ model groups, Model 3 should be regarded as the model with the worst attunement to the actual measured values. The RMSE values for this model were the highest in the given group, and the agreement with the $OAQI_{I}$ was at the lowest level of compliance. This model, like Model 4 and Model 5, was a hybrid model; however, a single sensor signal was used as an exponent of Steven’s power law. This shows that it is reasonable and fully justifiable to use gas sensor arrays to monitor air quality in terms of odor nuisance caused mostly by gaseous mixtures of complex composition. Their principle of operation is based on the overlapping of the detection ranges of individual sensors, which may help avoid problems associated with synergistic interactions between odorants (masking, neutralization, and amplification), which might significantly affect the signal from a single sensor.

Moreover, each of the models from the $S_{dB}$ group was characterized by a lower RMSE values than the most optimal model from the $S_{max}$ group (Model 2). This means that using a logarithmic method to extract signals from the sensor matrix which was meant to reflect the non-linear response to external stimuli of the human sense of smell (Weber–Fechner law), together with appropriate mathematical models, allows, with a higher accuracy, to predict the value of odor concentration.

In the last stage of the research, the constructed sensor matrix was used to control the odor air quality at the compost screening site in online mode (continuous monitoring). One mathematical model from each of the two proposed sensor signal acquisition groups was used to control the odor nuisance. Obviously, the models that were considered optimal on the basis of previous observations and measurements were applied, so they were Model 2 from the $S_{max}$ group and Model 5 from the $S_{dB}$ group. This phase lasted one week, and additional olfactometric measurements were taken daily in the vicinity of the sensor array for subsequent verification of the odor concentration values returned by the implemented models. Figure 9 shows the weekly course of changes in the odor concentration within the composting yard for both models and the values measured using field olfactometry, which was treated as the reference method.

As can be seen, the values of the odor concentration indicated by both models differ quite significantly. The values obtained by Model 2 practically do not fall below 20 $\mathrm{ou}/m^{3}$ in the entire period covered by this part of the research. Olfactometric measurements allow to conclude that the odor nuisance did not remain at such a high level all the time, but rather showed a variable character, which was probably related to the shift work mode at the municipal treatment plant and conducting compost screening processes in the afternoon or evening hours. This nature of the plant operation could also be confirmed by the odor concentration values obtained using Model 5. On the one hand, they showed a better match with the olfactometric measurements performed, and on the other, they allowed the identification of periods of increased odor nuisance, which usually occurred in the evening or at night. The overestimated odor concentration values obtained using Model 2 were likely due to the presence of odorless landfill gases, such as methane. Such gases will cause a significant increase in the maximum signal for a given sensor while not contributing to the odor nuisance. The phenomenon of saturation of the sensors might then occur, which will result in their transition to a non-linear operating range and can have a negative impact on the functioning of the developed model. Model 5 based on the dB scale appears to have less ability to overestimate the results which may mean that the dB scale along with a properly developed mathematical model used for continuous odor concentration monitoring at the municipal treatment plant is an appropriate approach.

It should additionally be noted that the values of the odor concentration indicated by the prepared models very rarely showed values below 3 $\mathrm{ou}/m^{3}$. This obviously could be due to inaccuracies, overestimations or underestimations of the values returned by the models. A solution improving the quality of sensor matrices and increasing the accuracy of implemented mathematical models could be the use of additional temperature, pressure and humidity sensors and modules that maintain stable working conditions inside the matrix’s measuring chamber. Another possible reason for the prepared models rarely indicating low odor concentration values could be the impact of facilities adjacent to the composting site, such as, for example, waste sorting buildings or green waste storage yards. Therefore, a comprehensive approach would seem to be to place several or more sensor arrays within the MSWTP so that odor air quality could be monitored throughout the plant. Establishing such a network of sensor meters would enable odor nuisance monitoring on a continuous basis and provide plant owners with information on the most odor sensitive points or processes. Moreover, measuring an odor emitting facility can be helpful if there is a lot of public pressure from residents living in the neighborhoods adjacent to the facility where the waste is stored and processed. Such a facility will be fully transparent to society, and the measurements taken will help verify the validity of incoming complaints and discourage people from overusing such odor nuisance countermeasures.

## 5. Conclusions

This paper proposes a new four-step odor air quality index ($OAQI_{I}$) that includes three odor parameters: intensity, hedonic tone and odor concentration. Due to the subjective assessment of odor parameters by humans, a gas sensor array was constructed which was adapted to determine the above-mentioned index in an instrumental manner. For this purpose, continuous measurements were carried out at the municipal solid waste treatment plant (MSWTP), using the developed sensor matrix, which was calibrated with field olfactometry. On the basis of collected data, five different mathematical models were tested to correlate the multidimensional output signal from the sensor array with the odor concentration of the sample. Two approaches were proposed for signals extraction from a sensor array: (i) the maximum signal value from an individual sensor—$S_{max}$, and (ii) the logarithm of the ratio of the maximum sensor signal value to its baseline which was determined by passing synthetic air through the sensors—$S_{dB}$. The second method used was to reflect the non-linear relationship between the measure of the olfactory stimulus and the response of the human sense of smell (Weber–Fechner law).

Standard approaches used for processing and analysis of data obtained from gas sensor matrix, such as multiple linear regression (MLR) or principal component regression (PCR), were applied to develop the models (Model 1 and Model 2). Additionally, models that resemble perceptual models of stimulus perception (Stevens’ law) were proposed (Model 3, Model 4 and Model 5). In these models, the empirical constant depending on the type of external stimulus was replaced by the signal from the sensor array after appropriate data processing. These three models represent a hybrid approach that combines the selling points of multidimensional sensor data analysis and the advantages of the psychophysical phenomenon for stimulus perception and processing in the human brain.

The research showed that the best model in terms of compatibility with the $OAQI_{I}$ class was a hybrid model (Model 5) in which the logarithmic approach to extract signals from the matrix was used. This model was prepared to reflect the effects of external stimulus on human perception (Weber–Fechner’s Law) in the form of a logarithmic way of sourcing signals from the matrix, the intensity of the perceived olfactory impression (Stevens’ law) in the form of a proposed equation, and the benefits of multivariate data analysis in the form of principal component analysis (PCA) and the use of first principal component ($PC_{1}$) as an exponent of a power in the prepared equation. The use of this model ensured a 98% consistency in classification into the classes of the proposed index. Model 3 was determined to be the worst in terms of agreement with the $OAQI_{I}$ class in both model groups ($S_{max}$ and $S_{dB}$). Admittedly, this was a hybrid model; however, it used single sensor signals without prior analysis and processing of the data acquired from the array.

The application of the developed model (Model 5) for the continuous monitoring of odorous air quality (odor concentration) in compost screening yard indicated that the *dB* scale was an appropriate choice. The *dB*-based model does not tend to overestimate the results. The overstating results for tha $S_{max}$ models are probably caused by the presence of gases at the site, which are odorless, but cause the applied sensors to react (e.g., methane). Presentation of the values of the signals from the sensor matrix in *dB* scale allows for lower variability in the case of the above mentioned situation.

## Figures and Tables

**Table 1:** The olfactory threshold for exemplary odorants [36,37,38,39].

Chemical Compound	Olfactory Threshold	Unit	Odor Character
Acetaldehyde	0.015–0.066	ppm	Fruity, apple
Formaldehyde	0.50–0.80	ppm	Pungent, suffocating
Acrolein	0.0036–0.16	ppm	Pungent, suffocating
Phenol	0.0056–0.040	ppm	Pungent
Hydrogen sulfide	0.41–0.81	ppb	Rotten eggs
Carbon disulfide	0.11–0.21	ppm	Rotten vegetables
Dimethyl sulfide	2.70–3.00	ppb	Rotten vegetables, garlic
Ammonia	1.50–5.20	ppm	Sharp, pungent
Methylamine	0.035–4.70	ppm	Fish, piscine
Dimethylamine	0.033–0.34	ppm	Fish, piscine
Acetone	13.00–42.00	ppm	Fruity, sweet
Acetic acid	0.0060–0.48	ppm	Vinegar
Acetonitrile	13–170	ppm	Etheric
Propionic acid	0.0057–0.16	ppm	Pungent
Acrylonitrile	1.60–17.0	ppm	Etheric
Sulfur dioxide	0.87–1.10	ppm	Pungent, suffocating
Ethyl mercaptan	0.0087–0.76	ppb	Rotten eggs, rotten cabbage
Nitrogen dioxide	0.12–0.36	ppm	Harsh
Pyridine	0.063–0.17	ppm	Strong sickening
Hexane	1.5–130	ppm	slightly disagreeable
Cyclohexane	2.5–25	ppm	Sweet
Toluene	0.33–2.50	ppm	Paint thinners
Benzene	2.70–12.00	ppm	Sweet, aromatic, gasoline

**Table 2:** Indicators and indexes used for evaluation of odor nuisance of exemplary industrial and municipal facilities.

Facility	Index	Scope of Research	References
WWTP ${}^{1}$	AOI SOI	Investigation of correlation between odors concentration measured by means of dynamic olfactometry (DO) and chromatographic GC-MS-FID analysis	[83]
MSWTP ${}^{2}$	SOEF OER OEF	A study of large anaerobic–aerobic treatment plant, identifying its odor sources, characterizing them in terms of odor concentration and emissions using dynamic olfactometry	[78]
Industrial park	OAI	13 potential odor emitting facilities; assessment of the odor annoyance using the residents as measuring tools—resident diary method	[84]
MSWTP	OER OEF	Mechanical and biological MSWTPs; calculation of OEFs, based on the results of olfactometric measurements, as a function of plants capacity which differ in constructional features, in type of treated waste and geographical locations in Italy	[79]
Compost facility	OAI	Assessment of odor annoyance generated by the composting facility and demonstration of the feasibility of gas sensor array to monitor the emission of the odorous substances	[85]
MSW landfills	SOER OER OEF	Estimation of odor emissions from landfills, focusing on the odor related to the emissions of landfill gas (LFG) from plant surface	[80]
MSW landfills	SOER OEF	Seven dimensionally different landfills, the odor concentration was calculated as the geometric mean of the odor threshold values of each panelist, using dynamic olfactometry	[81]
MRP ${}^{3}$	SOER OER OEF	Determination of odor nuisance from the rendering industry based on experimental data obtained by means of dynamic olfactometry, mass of processed material was used as “activity index” for OEF calculation	[82]
WWTP	OEF	Calculation of OEFs based on the results of olfactometric measurements that were carried out on a significant number of WWTPs, which differ in constructional features, in type of treated wastewater and in geographical locations in Italy; yearly treatment plant capacity was used as “activity index”	[86]
WWTP	OI	Investigation of the relationship odor index assessed by Japanese standard methods (triangle odor bag method) and odor concentrations measured with dynamic olfactometry	[87]
WWTP	OI	Relationship between odor concentrations emitted by WWTP assessed by Japanese standard methods and odor concentrations measured with dynamic olfactometry and compared to the measurement carried out by novel prototype of e-nose	[87]
WWTP	AOI SOI	Comparison and evaluation of the principal odor measurement methods (GC-MS, dynamic olfactometry, electronic nose) used to identify and characterize the odor emission from a WWTP with the aim of analyzing the weaknesses and strengths of the different techniques	[88]
Compost facility	OAV	The ability of *OAV* to predict odor concentrations during composting of six solid wastes and three digestates was evaluated, dynamic olfactometry and GC-MS were used as measurement methods	[89]
MSW landfills	OAV	Evaluation of odorant interaction effect to accurately estimate the contribution of odors, samples from a food waste treatment plant were analyzed by instrumental and olfactory methods, an odorant coefficient was proposed to assess the type and level of binary interaction effects based on *OAV* variation	[90]

^1^ Wastewater Treatment Plant, ^2^ Municipal Solid Waste Treatment Plant, ^3^ Meat Rendering Plant.

**Table 3:** Basic characteristics of selected chemical gas sensors.

Sensor Type	Manufacturer	Model	Detected Gases	Signal Processing
MOS ${}^{1}$	Figaro Engineering Inc. (Osaka, Japan)	TGS2602 [98]	hydrogen (1–30 ppm), toluene (1–30 ppm), ethanol (1–30 ppm), ammonia (1–30 ppm), hydrogen sulfide (0.1–3 ppm)	Voltage divider
MOS ${}^{1}$	Figaro Engineering Inc. (Osaka, Japan)	TGS2603 [99]	hydrogen (1-30 ppm), hydrogen sulfide (0.3–3.0 ppm), ethanol (1–30 ppm), methyl mercaptan (0.3–3.0 ppm), trimethyl amine (0.1–3.0 ppm)	Voltage divider
MOS ${}^{1}$	Figaro Engineering Inc. (Osaka, Japan)	TGS2612 [100]	methane (300–10,000 ppm), propane (300–10,000 ppm), ethanol (300–10,000 ppm), iso-butane (300–10,000 ppm)	Voltage divider
EC ${}^{2}$	Alphasense (Braintree, United Kingdom)	H2S-A4 [101]	hydrogen sulfide (limit of performance warranty 0–50 ppm)	I-U converter
EC ${}^{2}$	Alphasense (Braintree, United Kingdom)	NH3-B1 [102]	ammonia (limit of performance warranty 0–100 ppm)	I-U converter

^1^ Metal Oxide Semiconductor, ^2^ Electrochemical.

**Table 4:** Proposed odor air quality index ($OAQI_{I}$) scale and its parameters.

$OAQI_{I}$ Scale	Odor Intensity	Hedonic Tone	Proposed $C_{\mathrm{OD}}$ Range
0—very good	non-perceptible and very weak	neutral and slightly unpleasant	$0<C_{OD}\leq 3$
1—moderate	weak	moderately unpleasant	$3<C_{OD}\leq 10$
2—bad	distinct and strong	very unpleasant	$10<C_{OD}\leq 60$
3—very bad	very and extremely strong	extremely unpleasant	$C_{OD}>60$

**Table 5:** Accordance and RMSE for prepared models.

Signals Features	Model	RMSE	Accordance
$S_{max}$	Model 1	10.302	0.70
	Model 2	8.092	0.85
	Model 3	67.973	0.65
	Model 5	7.399	0.75
$S_{dB}$	Model 1	5.754	0.75
	Model 2	5.420	0.85
	Model 3	7.120	0.70
	Model 4	3.667	0.90
	Model 5	4.220	0.98

## Data Availability

Not applicable.

## Sample Availability

Samples of the compounds are not available from the authors.
